# The Narrow Corridor of Heartfelt Leadership: Social and Emotional Leadership Practices in Bureaucratic School Cultures

**DOI:** 10.3390/bs15101316

**Published:** 2025-09-26

**Authors:** Sevgi Yıldız

**Affiliations:** Faculty of Education, Ordu University, Ordu 52200, Türkiye; yldzsvg@gmail.com

**Keywords:** heartfelt leadership, social and emotional leadership, bureaucratic school culture, emotional intelligence, qualitative research

## Abstract

This qualitative study examines how school administrators enact “heartfelt leadership”—a socially and emotionally attuned form of leadership—within Türkiye’s bureaucratic school cultures. Using semi-structured interviews with six administrators and six teachers across primary, secondary, and high school, we employed a basic qualitative design with maximum-variation sampling. Thematic content analysis yielded four themes: (1) principals acknowledge the humanity of their teachers; (2) principals prioritize relationships and go beyond formal duties; (3) bureaucracy constrains but does not fully silence heartfelt leadership; and (4) heartfelt leadership fosters motivation, resilience, and retention. Heartfelt leadership was marked by empathy, recognition of significant moments, and proactive care that extends beyond job descriptions, cultivating trust, motivation, and commitment. Yet rigid procedures and centralized decision-making limited leaders’ autonomy and responsiveness. No consistent gender differences emerged among principals, because all participating teachers were female; therefore, cross-gender comparisons among teachers were not possible. Theoretically, the study bridges emotional-intelligence and bureaucratic-organization scholarship, showing how relational leadership can be sustained in centralized systems through micro-level strategies. Empirically, it broadens global leadership discourse by examining emotional leadership in a non-Western, bureaucratic context. Practically, findings suggest embedding social and emotional competencies in leadership preparation and enabling greater discretionary authority for responsive, human-centered school leadership.

## 1. Introduction

Throughout this article, school leadership’ is used as the general descriptor, and ‘school administrators’ is used to refer to formal managerial roles within that leadership context. In the evolving discourse of educational leadership, social and emotional competencies have gained increasing recognition as essential attributes for effective school leaders (Fullan, 2020; Goleman, 1995; Brackett et al., 2011). Within this paradigm, heartfelt leadership—defined in this study as leadership grounded in empathy, relational trust, and authentic care—extends beyond managerial responsibilities to address the deeper emotional and relational needs of teachers and students. Leaders who embody this approach function not only as administrators but also as emotional anchors, fostering psychologically safe environments that sustain professional motivation, collaboration, and well-being (Leithwood & Beatty, 2008; Baxter & Ehren, 2023). To complement foundational work (e.g., Goleman, 1995), recent scholarship indicates that socially and emotionally attuned leadership is associated with greater psychological safety, lower burnout, and stronger teacher commitment (Hascher & Waber, 2021; Dreer, 2023; Harris & Jones, 2023). International evidence further underscores the policy relevance of social and emotional skills for schooling (OECD, 2021).

Heartfelt leadership shares significant similarities with the ethic of care tradition in educational leadership. Like leaders operating with an ethic of care, heartfelt leaders prioritize empathy, compassion, trust, and responsiveness to the needs of others (Noddings, 1984, as cited in Arar et al., 2016). Administrators leading with an ethic of care lead with empathy and compassion. They are responsive to stakeholder needs, place immense value in trust and relationships, and empower students (and others) to be their full, authentic selves (Arar et al., 2016). Both approaches emphasize relational authenticity and the moral imperative to recognize and affirm the humanity of teachers and students. While ethic of care leadership provides the philosophical foundation, heartfelt leadership in this study operationalizes these principles within the bureaucratic school context in Türkiye, uniquely adapting them to centralized and proceduralized educational environments and showing how leaders enact care-driven practices despite systemic constraints (Arar et al., 2016; Stefkovich & Begley, 2007; Tenuto & Gardiner, 2018).

However, enacting heartfelt leadership in highly bureaucratic education systems presents inherent challenges. In Türkiye, the education system is characterized by centralized decision-making, hierarchical authority structures, and rigid procedural frameworks (Bush, 2020; Hoy & Miskel, 2013). These bureaucratic conditions create a *narrow corridor* in which school leaders must balance human-centered responsiveness with the procedural demands of compliance. This tension echoes Weber’s (1922/1978) theorization of bureaucratic “institutional tightness” and reflects the dilemmas leaders face in sustaining emotional engagement under structural constraint.

While international research demonstrates that emotionally attuned leadership enhances teacher motivation, reduces burnout, and strengthens organizational trust (Leithwood & Beatty, 2008; Maslach & Leiter, 2016), there is a paucity of empirical studies examining how such practices operate within centralized, bureaucratic systems—particularly in non-Western contexts. Türkiye presents a compelling case study due to its strong bureaucratic tradition, centralized governance, and ongoing debates surrounding leadership autonomy in educational policy and practice. This study addresses the following research questions:

1. How do school administrators and teachers conceptualize social and emotional leadership in their schools?
2. What specific practices reflect heartfelt leadership in bureaucratic school settings?
3. What bureaucratic constraints hinder the enactment of heartfelt leadership?
4. What outcomes are associated with heartfelt leadership for teachers, students, and the school community?

By addressing these questions, the study makes three contributions. Theoretically, it integrates emotional intelligence theory with bureaucratic organizational frameworks, offering a nuanced understanding of leadership agency under structural constraints. Empirically, it provides qualitative evidence from a non-Western bureaucratic education system—an underrepresented context in the global leadership literature. Practically, it identifies actionable strategies for embedding emotional responsiveness into school leadership without compromising procedural accountability. In doing so, the study positions heartfelt leadership as a culturally grounded construct that captures the complex interplay between emotional responsiveness and bureaucratic control in Türkiye’s public schools.

## 2. Conceptual Framework

### 2.1. Social and Emotional Leadership and the Heartfelt Leadership Lens

Social and emotional leadership is grounded in a leader’s capacity to perceive, interpret, and respond to emotion—both one’s own and others’—through self-awareness, empathy, emotion regulation, and relationship management (Goleman, 1995; Brackett et al., 2011; Mayer et al., 2016). In school settings, these competencies translate into attentiveness to teachers’ psychological needs, the cultivation of a psychologically safe climate, and the recognition of individual circumstances in both professional and personal domains. A substantial body of research links such leadership to higher job satisfaction, reduced burnout, and stronger organizational commitment (Leithwood & Beatty, 2008; Day et al., 2001). By building trust-based relationships and demonstrating authentic care, leaders help reduce professional isolation, heighten teachers’ sense of belonging, and foster collaborative work practices (Bryk & Schneider, 2002; Edmondson, 1999; Leithwood & Beatty, 2008).

This study introduces heartfelt leadership as a locally grounded articulation of social and emotional leadership that emphasizes warmth, relational depth, and human-centered decision-making. Rooted in the foundational work of Goleman (1995) on emotional intelligence, Leithwood’s transformational leadership framework, and Brackett’s social-emotional learning model, heartfelt leadership prioritizes empathy, responsiveness to teachers’ lived realities, and the consistent recognition of both personal and professional milestones.

Within this landscape, heartfelt leadership is conceptualized as a context-attuned articulation of social and emotional leadership, in which warmth, relational depth, and human-centered decision-making are foregrounded. Through a synthesis of emotional-intelligence competencies (Goleman, 1995; Brackett et al., 2011) with a transformational orientation to people and culture, responsiveness to teachers’ lived realities and the consistent recognition of personal and professional milestones are emphasized. In Türkiye’s highly centralized system, heartfelt leadership is not a formal requirement but an intentional practice through which principals navigate procedural constraints while sustaining emotional connection with staff; it is not merely a skill set but a value-driven orientation that humanizes schooling. Contemporary syntheses connect leaders’ emotional competencies with school culture, teacher commitment, and retention, providing updated support for this framework (Leithwood et al., 2020; Gómez-Leal et al., 2022; Floman et al., 2024).

### 2.2. Bureaucratic Culture in Schooling: The Turkish Governance Context

Bureaucratic culture—rooted in Weber’s classical organizational theory—is characterized by formal rules, hierarchical structures, and rationalized procedures intended to ensure consistency and control (Weber, 1922/1978). In schools, this typically manifests as centralized authority, standardized communication channels, and top-down decision-making (Hoy & Miskel, 2013; Bush, 2020). In Türkiye, the governance of education is explicitly centralized by statute and policy: the Ministry of National Education (MoNE) directly regulates curriculum, teacher appointments, administrator assignments, and financial management, while provincial and district directorates act as intermediaries within a vertical communication structure where decision-making flows predominantly from top to bottom (OECD, 2020; Eurydice, 2025).

Although such arrangements can promote equity and procedural fairness, they also tend to narrow the space for emotional expression and flexibility in day-to-day leadership. In Hoy and Sweetland’s (2001) terms, bureaucratic arrangements can become coercive—rather than enabling—thereby dampening initiative, discretionary judgment, and the responsiveness that relational leadership requires (see also Weber, 1922/1978; Leithwood & Beatty, 2008). In practice, attributes such as empathy, openness, and compassion risk being interpreted as “soft” or even counterproductive in rigid procedural environments; the enactment of social and emotional leadership may therefore depend more on the personal disposition and moral purpose of the principal than on institutional encouragement.

### 2.3. How Heartfelt Leadership Operates Within Bureaucracy: Mechanisms, Risks, and Payoffs

Heartfelt leadership is interdependent with social-emotional competencies. Empathy, active listening, and timely support provide the practical foundation for heartfelt behaviors, yet bureaucratic “tightness” can suppress these behaviors by limiting discretionary authority and slowing action through procedural requirements (Bakker & Demerouti, 2017; Hoy & Sweetland, 2001). When heartfelt practices are successfully integrated into bureaucratic contexts, relational trust, collegial communication, and teachers’ emotional well-being are strengthened—factors empirically associated with higher engagement and reduced turnover intentions (Leithwood & Beatty, 2008; Skaalvik & Skaalvik, 2017). Conversely, strict rule adherence in the absence of relational flexibility is associated with diminished morale, attenuated responsiveness to individual needs, and heightened risk of burnout, particularly in high-demand, low-autonomy settings (Bakker & Demerouti, 2017; Skaalvik & Skaalvik, 2017).

In short, heartfelt leadership operates as a relational counterbalance to structural rigidity: it preserves the human texture of school life while working within the system’s constraints, and it clarifies the conditions under which emotionally attuned leadership can be both ethically grounded and organizationally effective in Türkiye’s centralized public schools.

## 3. Methodology

This study employed a basic qualitative research design to explore how school administrators and teachers perceive and enact heartfelt leadership within bureaucratic school cultures in Türkiye. This design was selected because it allows for an in-depth examination of participants’ lived experiences and the meanings they ascribe to them, without being bound to the rigid procedures of other qualitative traditions (Merriam & Tisdell, 2016).

### 3.1. Participants

A total of twelve participants—six school administrators and six teachers—were selected through maximum variation sampling to ensure diversity in school level, professional role, gender, and years of experience. This sampling strategy allowed for the identification of shared patterns as well as variations across different institutional and personal backgrounds (Patton, 2015). All participants worked in public schools affiliated with the Ministry of National Education during the 2024–2025 academic year. All participating teachers were female. Although maximum variation sampling was employed, only female teachers volunteered and consented to participate during the data collection process. Of the school administrators, four were male and two were female. The participants represented educational settings across preschool through K–12 levels. A broader pool of eligible principals and teachers was approached through purposive contacts aligned with the maximum-variation strategy; twelve participants (six principals, six teachers) consented and met inclusion criteria for participation.

### 3.2. Data Collection

Data were collected through semi-structured interviews designed to elicit rich, detailed accounts of participants’ experiences with social and emotional leadership in their schools. The interview protocol included open-ended questions on conceptualizations of heartfelt leadership, concrete leadership practices, bureaucratic constraints, and perceived outcomes. Each interview lasted between 45 and 70 min and was conducted face-to-face in a setting chosen by the participant to ensure comfort and confidentiality. All interviews were audio-recorded with informed consent and subsequently transcribed verbatim. Examples of semi-structured interview prompts used in the study:

- What place do emotion and relationships hold in your own understanding of leadership?
- Have you experienced a leadership moment you would describe as heartfelt? How would you describe it?
- Do you believe there is a conflict between bureaucratic school culture and heartfelt (social and emotional) leadership?
- To what extent does the education system enable—or constrain—school leaders to lead from the heart?
- Which attitudes or behaviors do you find most valuable in your relationship with your administrator?
- Can you describe a time when you felt your principal offered heartfelt support?
- How do bureaucratic practices within school culture affect teacher–administrator relationships?
- In your view, what specific strategies can administrators use to enact heartfelt leadership more consistently?

### 3.3. Data Analysis

The data were analyzed using thematic content analysis (Braun & Clarke, 2006) with the support of NVivo 12 software (trial version, Lumivero, Melbourne, Australia). The analysis proceeded in three iterative phases: (1) initial coding to identify meaningful units of data; (2) clustering of codes into categories that reflected broader concepts; and (3) development of overarching themes aligned with the research questions. Themes were refined through constant comparison across cases, ensuring that they accurately represented the range of perspectives. Memos and reflexive notes were kept throughout the process to enhance analytical rigor.

### 3.4. Trustworthiness

To ensure the credibility of findings, member checking was conducted by sharing preliminary themes with selected participants for feedback and verification. Peer debriefing sessions were held with two experts in educational leadership to discuss coding decisions and thematic interpretations. An audit trail documenting methodological decisions and analytical steps was maintained to enhance dependability and confirmability (Guba & Lincoln, 2018).

### 3.5. Ethical Considerations

Ethical approval for the study was obtained from the relevant institutional review board. Participants were provided with an information sheet detailing the study’s aims, procedures, and their rights, including the right to withdraw at any stage without penalty. Informed consent was obtained in writing, and all identifying details were anonymized to protect confidentiality.

## 4. Findings

The findings of this study are based on in-depth, semi-structured interviews conducted with teachers and school administrators. Coding and theme development were facilitated by NVivo 12, which enabled systematic organization of the data, supported the identification of recurrent themes, and enhanced the rigor of the thematic content analysis. The analysis yielded four overarching themes: (1) Understanding Social and Emotional Leadership, (2) Practices of Leading from the Heart, (3) Bureaucratic Barriers to Heartfelt Leadership, and (4) Perceived Outcomes of Heartfelt Leadership. Each theme is presented with sub-themes, direct quotations, and interpretive commentary, followed by a brief thematic summary linking the results to broader literature. With respect to gender, no consistent differences emerged among principals’ accounts, and because all participating teachers were female, cross-gender comparisons among teachers were not possible.

The thematic coding framework, generated using NVivo 12 software, is presented in Table 1. This visual representation maps the four main themes and their related sub-themes, illustrating the relationships between the conceptual categories and supporting quotes that emerged during analysis.

### 4.1. Theme 1: Principals Acknowledge the Humanity of Their Teachers

Participants consistently described heartfelt leadership as a practice where principals recognize teachers not only as professionals but also as human beings with emotional and personal needs. Empathy, emotional awareness, and human-centered decision-making were identified as integral to fostering trust, building a positive school climate, and sustaining professional relationships. This perspective aligns with Goleman’s (1995) emotional intelligence model, which emphasizes self-awareness and relational management, and with Leithwood and Beatty’s (2008) argument that emotional engagement enhances teacher commitment.

### 4.2. Sub-Theme 1.1: Empathy and Emotional Awareness

Empathy was perceived as the cornerstone of heartfelt leadership. Leaders who recognized and responded to emotional cues—often without verbal disclosure—were seen as making a significant impact on teachers’ daily experiences. One teacher (T1) reflected:

“A principal should be present in both good and bad times, understanding what we go through and making us feel valued. My current principal notices when I am upset, even before I say anything. Once, during a particularly stressful week, he said, ‘I’ll cover your class for the last period today, go get some rest.’ That moment stayed with me. It wasn’t written in any job description; it was simply human care.” Another teacher (T3) recalled:

“Last year, I had a serious health issue. Before I even asked, my principal rearranged my schedule so I could go to the hospital regularly. He called me in the evenings to check on my recovery, not to ask about school work. That’s when I realized leadership is not only about rules and performance—it’s about people.”

These accounts support Leithwood and Beatty’s (2008) argument that authentic care reduces stress and fosters loyalty. Recent research confirms that relational trust, reinforced by emotional attunement and leader visibility, acts as a protective factor for teacher well-being (Baxter & Ehren, 2023; Jack, 2023). Such practices exemplify what Hochschild (1983/2012) terms “emotional labor,” voluntarily performed as a leadership choice rather than a bureaucratic duty.

### 4.3. Sub-Theme 1.2: Recognition of Significant Moments

Recognition of both personal and professional milestones emerged as another defining feature of heartfelt leadership. Teachers described how leaders who acknowledged meaningful moments fostered a sense of belonging and validation. One participant (T2) shared:

“Our principal remembers birthdays, work anniversaries, and even small successes we have in class. When I won a district-level teaching award, he arranged a surprise celebration. It made me feel like my effort mattered not just to me, but to the whole school.”

Another teacher (T5) noted: “I once mentioned that it was my daughter’s first day at kindergarten. That afternoon, my principal asked how it went. I was surprised he remembered—it showed me he listens even to small details.” From a theoretical perspective, this resonates with Deci and Ryan’s (2000) self-determination theory, which highlights acknowledgment as a driver of intrinsic motivation, and with Skaalvik and Skaalvik’s (2017) findings on personal validation as a buffer against stress. In bureaucratic environments often criticized for impersonality (Hoy & Miskel, 2013), such acts humanize leadership and build collective efficacy (Bass & Riggio, 2006).

The findings on how participants conceptualize social and emotional leadership reveal that this understanding is not merely a set of values but is also translated into concrete practices. Therefore, the next section focuses on how the ‘heartfelt leadership’ approach is enacted in the daily life of schools.

### 4.4. Theme 2: Principals Prioritize Relationships and Go Beyond Formal Duties

Heartfelt leadership was described not only as an attitude but as a set of practices that consistently prioritized relationships and extended beyond formal job requirements. These practices included providing resources, personal involvement in teachers’ needs, and informal relational gestures that cultivated belonging and trust. Such actions reflected a genuine commitment to teachers’ well-being and students’ success. This finding resonates with the literature on proactive and relational leadership (Day et al., 2001; Leithwood & Beatty, 2008), where leadership effectiveness is linked to relational attentiveness and going “above and beyond” in everyday and exceptional moments.

### 4.5. Sub-Theme 2.1: Providing Resources and Support

Participants described heartfelt leaders as those who took proactive steps to provide material, moral, and logistical support—even when formal budgets or procedures fell short. These acts were experienced not as peripheral “niceties” but as core to the leadership role because they directly reduced teachers’ day-to-day strain and enabled instructional improvement. In Job Demands–Resources (JD–R) theory, leaders’ tangible help functioned as an organizational resource that buffered high job demands and sustained motivation (Bakker & Demerouti, 2017). Teachers frequently framed these moments through the lens of felt support—“my work and well-being matter here”—which closely aligns with the perceived organizational support literature (Eisenberger et al., 1986; Rhoades & Eisenberger, 2002). One teacher (T2) recounted:

“Last year, a group of students wanted to participate in a national competition but couldn’t afford the entry fees. Our principal didn’t wait for approval or budget allocation—he personally paid for them, saying it was an investment in their future. That made me realize leadership is not just about administration—it’s about putting your heart into it.”

A principal (A4) explained the rationale behind such “above-and-beyond” actions: “A teacher needed materials for a project, but the school budget was empty. I drove to the city myself and bought the supplies because I knew the project would broaden the students’ horizons.” These examples mirror what Hoy and Sweetland (2000) describe as enabling bureaucracy—leaders flex the system (without violating it) to remove obstacles so teachers can do their best work. They also align with evidence that principals’ strategic resourcing and problem-solving agility are consistent features of effective school leadership (Robinson et al., 2008; Grissom et al., 2021). Practically, such support not only equips classrooms but signals trust in teachers’ professional judgment, a known driver of engagement and discretionary effort (Bass & Riggio, 2006; Leithwood & Beatty, 2008).

### 4.6. Sub-Theme 2.2: Building Informal Connections

Alongside resource provision, heartfelt leadership was also “felt” in the micro-interactions of everyday school life—greetings at the door, unhurried chats in the staffroom, remembering the details that matter. Participants consistently linked these informal ties to a stronger sense of belonging and openness. This maps onto the notion of relational trust (Bryk & Schneider, 2002) and psychological safety (Edmondson, 1999, 2018): when teachers experience the leader as approachable and attuned, they are more willing to surface problems early, share half-formed ideas, and collaborate across boundaries. A principal (A3) described this intentional informality:

“I make it a habit to sit in the teachers’ lounge, have tea, and talk about their lives, not just work. I know whose child is preparing for an exam, who is planning a holiday. These moments create bonds that no meeting agenda can.”

A teacher (T1) echoed the effect: “Every morning, our principal greets us at the door and asks how we’re doing. Even if my day starts badly, this lifts my mood. You feel like a person first, then a teacher.” Beyond “being nice,” these routines cultivate high-quality leader–member exchange (LMX), which research associates with greater commitment and performance (Graen & Uhl-Bien, 1995). They also create a climate where small wins accumulate (Weick, 1984): minor yet repeated affirmations gradually shift norms toward candor, help-seeking, and collective efficacy—particularly valuable in bureaucratic settings that can otherwise feel impersonal (Hoy & Miskel, 2013).

Taken together, resource provision and informal connection-building constitute complementary dimensions of heartfelt leadership. The former addresses structural and material needs, while the latter strengthens relational and emotional bonds. Both operate as reinforcing mechanisms: tangible support enhances trust in leadership, while relational closeness increases the willingness to collaborate and innovate. In bureaucratic school cultures, where impersonal rules often dominate, these intertwined practices carve out a relational space that sustains teacher motivation, well-being, and commitment to the school community.

While the daily practices of heartfelt leadership create a strong sense of belonging and motivation for teachers and administrators, these positive actions often come into conflict with the bureaucratic structure of the school system and are constrained by various limitations. The following theme examines how such bureaucratic barriers are reflected in leadership practices.

### 4.7. Theme 3: Bureaucracy Constrains but Does Not Fully Silence Heartfelt Leadership

Although participants aspired to enact heartfelt leadership, they consistently emphasized that the bureaucratic structure of the education system imposed rigid constraints on their practices. Centralized policies, procedural rules, and hierarchical decision-making often clashed with their relational values, producing a tension between compliance and care. Yet, participants also described moments where they sought creative ways to circumvent or soften bureaucratic barriers to sustain relational leadership. This aligns with Hoy and Miskel’s (2013) notion of the “institutional tightness” of bureaucratic organizations while illustrating how principals still strive to preserve care-driven practices within these limits.

### 4.8. Sub-Theme 3.1: Procedural Constraints

Teachers and principals alike reported that even well-intentioned acts of care or support were frequently blocked—or at least delayed—by formal procedures. While designed to ensure fairness and accountability, these procedures often generated frustration and hindered timely responsiveness. A teacher (T3) shared a personal experience:

“My principal wanted to adjust my timetable because of a family situation, but later told me the district didn’t approve. He said, ‘I’m sorry, my hands are tied.’ It’s not that he didn’t care—he did—but good intentions aren’t always enough here.”

A principal (A2) recounted a similar case:

“I tried to get a scholarship for a student in need. Everything was ready—the sponsors, the money—but the paperwork delayed it for months. The student’s morale dropped in the meantime, and I felt powerless. Bureaucracy sometimes makes us late to the point where the help is no longer as meaningful.”

These accounts mirror Weber’s (1922/1978) theorization that rule-bound systems often sacrifice timeliness and human responsiveness in favor of procedural uniformity. In the context of heartfelt leadership, such delays not only undermine the effectiveness of supportive actions but also diminish their symbolic and emotional resonance. This aligns with research on “administrative lag” (Firestone, 2014), which shows that delayed responsiveness in high-demand contexts can erode trust and engagement over time.

### 4.9. Sub-Theme 3.2: Centralized Decision-Making and Lack of Autonomy

Another recurrent theme was the limited autonomy school leaders had in making decisions that directly affected their communities. Many described feeling like “implementers” rather than “leaders,” with key decisions—sometimes even on minor matters—made at higher administrative levels. A teacher (T4) illustrated this frustration:

“We wanted a library, and the whole school was excited. But the provincial office decided on a sports hall instead. Our principal agreed with us but said, ‘There’s nothing we can do; the decision came from above.’ That killed the momentum.”

A principal (A5) echoed this sense of constraint: “Even minor changes—like repainting a classroom—require multiple approvals. By the time you get the green light, the opportunity or enthusiasm is gone. Creativity is hard to sustain in such a system.” The lack of local decision-making capacity not only restricts leaders’ ability to respond quickly but also undermines the relational capital they build with staff and students. As Leithwood and Beatty (2008) note, autonomy is a critical enabler of relational leadership; without it, leaders risk being perceived as distant or ineffective, even when they share the community’s priorities. Recent research underscores that excessive centralization can erode school leaders’ problem-solving agility, limiting their capacity to address emergent needs in a timely and context-sensitive manner (Glatter, 2020). In highly bureaucratic systems, leaders often become “policy conduits” rather than proactive change agents, a role misalignment that constrains innovation and weakens relational trust.

Bureaucratic barriers constrain heartfelt leadership through two interlocking mechanisms: procedural rigidity and centralized authority. Procedural rigidity delays or dilutes emotionally responsive actions, while centralized authority limits leaders’ discretion to tailor solutions to local needs. These constraints weaken the relational impact of leadership, even when leaders possess strong social-emotional competencies and the will to act. Addressing this tension requires systemic reforms—such as decentralizing certain decision-making powers and streamlining approval processes—to expand leaders’ capacity for timely, contextually attuned, and emotionally grounded leadership practices.

Despite these bureaucratic constraints, participants emphasized that heartfelt leadership practices have not entirely disappeared; rather, under certain conditions, they continue to generate meaningful effects. The final theme explores the perceived outcomes of this leadership approach for teachers, students, and the overall school climate.

### 4.10. Theme 4: Heartfelt Leadership Fosters Motivation, Resilience, and Retention

Participants consistently associated heartfelt leadership with positive professional and organizational outcomes. Teachers reported enhanced motivation, a stronger sense of collaboration, reduced burnout, and an increased commitment to remain in the profession. These outcomes illustrate how heartfelt leadership not only supports emotional well-being but also strengthens resilience and teacher retention in the face of systemic pressures. This finding is consistent with Brackett et al. (2011), who argue that emotionally attuned leadership fosters psychological safety, trust, and discretionary effort among staff.

### 4.11. Sub-Theme 4.1: Motivation and Engagement

Teachers reported higher levels of motivation and professional engagement when their leaders recognized their contributions, trusted their professional judgment, and supported their initiatives. Such motivation often translates into a willingness to assume additional responsibilities, experiment with innovative practices, and invest personal resources in their work. A teacher (T1) recalled:

“After organizing the science fair, my principal didn’t just say ‘thank you’—he gathered the whole staff, explained what I had done in detail, and expressed how proud he was. I felt so valued that I immediately volunteered for another project.”

Another teacher (T2) added: “When my principal trusts me, I work harder, not because I have to, but because I don’t want to disappoint them. Trust is a powerful motivator.” From the leadership perspective, principals recognized this reciprocal dynamic. A principal (A1) reflected: “Motivated teachers innovate more. I’ve seen teachers buy materials from their own pockets, stay after school to prepare lessons, simply because they know I have their back.” These accounts illustrate the mutually reinforcing cycle between leader trust and teacher engagement—a relationship extensively documented in transformational leadership literature (Bass & Riggio, 2006). In this framing, heartfelt leadership operates as a catalyst for intrinsic motivation, activating teachers’ professional agency and sustaining high levels of commitment even in resource-constrained environments.

### 4.12. Sub-Theme 4.2: Reduced Burnout and Emotional Resilience

Several participants emphasized heartfelt leadership’s role in reducing burnout and enhancing teachers’ emotional resilience. Leaders achieved this by adjusting workloads, offering empathetic listening, and providing meaningful recognition. A teacher (T4) recounted: “I was burned out and on the verge of quitting. My principal reassigned some classes so I could rest. It made a huge difference—not just physically but emotionally.” Another teacher (T5) reflected: “After 20 years in the classroom, my energy was low. But my principal encouraged me, gave me meaningful responsibilities, and treated me like my experience mattered. I felt young again.” From a leadership perspective, principals viewed this as a deliberate and strategic responsibility. As one principal (A3) put it: “When teachers start to burn out, the leader’s role is to be there for them—sometimes by giving them time, sometimes just by listening. That can reignite their passion for teaching.”

These narratives align with the Job Demands–Resources model (Bakker & Demerouti, 2017), which posits that emotional support from leaders functions as a critical resource buffering the negative effects of high job demands. Recent studies confirm that leaders who actively prioritize emotional connection and supportive dialogue can significantly reduce teacher burnout and enhance adaptive coping strategies in high-pressure educational settings (Floman et al., 2024; Yong & Zhang, 2025; Zhou et al., 2025). In this light, emotional engagement emerges not as a peripheral “soft skill” but as a strategic leadership practice essential for sustaining teacher well-being, retention, and institutional stability.

The perceived outcomes of heartfelt leadership reveal its dual role as both an emotional and structural resource in schools. By cultivating trust, recognizing contributions, and buffering against burnout, heartfelt leaders create conditions where teachers can thrive professionally and personally. These outcomes are particularly significant in bureaucratic systems where autonomy is limited; here, emotional leadership acts as a counterbalance to structural rigidity, preserving teacher morale and maintaining a resilient, engaged workforce.

## 5. Discussion

Beyond merely identifying bureaucratic barriers, this study contributes by illustrating the strategies principals employed to navigate and circumvent these constraints. Participants described practices such as flexible interpretation of formal rules, drawing on personal resources, and investing time in informal relational encounters with teachers. These practices enabled them to maintain a sense of relational authenticity even within centralized and highly proceduralized structures. Highlighting these adaptive strategies demonstrates that heartfelt leadership is not simply hindered by bureaucracy but rather reshaped through leaders’ agency in finding creative pathways to enact care-driven practices. This nuance advances literature by showing how relational leadership can persist, and even thrive, under bureaucratic conditions. These results are consistent with recent post-2020 scholarship indicating that emotionally attuned, relationship-focused leadership bolsters psychological safety, buffers burnout, and sustains engagement (Hascher & Waber, 2021; Dreer, 2023; Harris & Jones, 2023), and align with international evidence on social-emotional skills and schooling (OECD, 2021).

The findings of this study demonstrate that heartfelt leadership—anchored in social and emotional competencies—plays a critical role in shaping the school climate, fostering teacher motivation, and promoting overall organizational well-being. The themes identified—empathy and emotional awareness, recognition of significant moments, tangible acts of care, informal connections, and navigating bureaucratic constraints—extend and nuance the existing body of literature on emotional intelligence and educational leadership (Goleman, 1995; Leithwood & Beatty, 2008; Fullan, 2020).

First, empathy and emotional awareness emerged as central to participants’ conceptualization of effective leadership. Teachers valued leaders who could attune themselves to subtle emotional cues and respond proactively to both personal and professional needs. This aligns with Goleman’s (1995) framework, which identifies empathy as a cornerstone of emotional intelligence and a prerequisite for trust-building. In contexts where the demands of teaching are high and emotional strain is common, leaders who notice distress and act with compassion can serve as stabilizing forces. In this study, examples such as rearranging schedules for health reasons or anticipating needs without formal requests illustrate what Hochschild (1983/2012) terms emotional labor, performed here as a deliberate leadership choice rather than an administrative requirement.

Second, recognition of significant moments was perceived as more than symbolic; it was a tangible affirmation of teachers’ professional worth and personal identity. Previous research indicates that recognition fosters intrinsic motivation and organizational commitment (Deci & Ryan, 2000; Skaalvik & Skaalvik, 2017). Participants described how celebrations of milestones, whether birthdays or professional achievements, fostered a sense of belonging. In bureaucratic contexts often criticized for their impersonality (Hoy & Miskel, 2013), such acts represent a form of resistance—humanizing the institutional culture through relational gestures.

Third, tangible acts of care and informal connections reflect what Sergiovanni (1992) calls moral leadership, where the leader prioritizes human relationships over procedural compliance. Providing material resources from personal funds or dedicating time to informal interactions counters the transactional tendencies of bureaucratic systems. This finding is consistent with Leithwood et al.’s (2020) assertion that authentic relational engagement strengthens teacher resilience and job satisfaction. Moreover, the fact that principals in this study often engaged in such actions voluntarily highlights the discretionary nature of heartfelt leadership in highly centralized education systems.

However, the bureaucratic barriers identified in this study resonate with Bush’s (2020) critique of centralized governance structures, where decision-making is removed from local contexts. Procedural constraints, hierarchical approval processes, and rigid policy frameworks were seen to limit the scope of leaders’ autonomy, even when intentions were aligned with teachers’ needs. For example, delays in resource allocation or inflexibility in scheduling illustrate how systemic rigidity undermines the immediacy and responsiveness required for heartfelt leadership to flourish. This structural tension mirrors Hoy and Sweetland’s (2001) observation that bureaucratic control often conflicts with enabling leadership practices.

Finally, the study underscores heartfelt leadership as a protective factor against burnout. Teachers consistently linked acts of empathy, trust, and support to renewed motivation, reduced emotional exhaustion, and increased willingness to innovate. This finding is supported by Maslach and Leiter’s (2016) burnout framework, which identifies social support as a key buffer in high-stress professions. In particular, the provision of meaningful responsibilities, adjustment of workloads, and public recognition of contributions were reported to restore teachers’ sense of professional efficacy—a dynamic also observed by Skaalvik and Skaalvik (2017) in their research on teacher resilience.

In sum, the interplay between heartfelt leadership and bureaucratic culture presents both opportunities and constraints. While individual leaders can enact practices that humanize school life, their capacity to do so is mediated by the structural and procedural realities of centralized systems. This duality suggests that sustaining heartfelt leadership requires not only the personal competencies of individual leaders but also systemic reforms that grant greater autonomy and flexibility at the school level.

## 6. Limitations

This study has certain limitations that should be considered when interpreting the results. The findings are based on a relatively small sample of teachers and principals from a specific geographical region in Türkiye, which may limit the generalizability of the results to other contexts. Additionally, all participating teachers in this study were female, which limits the gender representativeness of the sample. The reliance on self-reported data through interviews may have introduced bias, as participants might have presented socially desirable responses. Finally, while the qualitative approach allowed for an in-depth exploration of experiences, it does not provide statistical evidence of causal relationships between heartfelt leadership practices and educational outcomes.

## 7. Conclusion and Recommendations

Conclusion: This study shows that heartfelt leadership—grounded in social and emotional competencies—is essential for sustaining teacher motivation, trust, and collaboration. Within Türkiye’s highly bureaucratic system, such practices often rely on principals’ personal initiative and emotional commitment rather than systemic support. Acts of empathy, recognition of significant moments, and tangible gestures of care were central to a positive climate in which teachers feel valued both professionally and personally. At the same time, rigid procedures and centralized decision-making limit leaders’ autonomy and responsiveness, creating a persistent tension between relational values and bureaucratic demands. The significance of heartfelt leadership lies not only in its positive outcomes but also in the strategies leaders develop to sustain care and empathy despite systemic obstacles.

Practical recommendations: Leadership preparation should embed social and emotional competencies and reflective practice; policy should legitimize relational work by protecting time for informal check-ins and recognition routines; where permissible, micro-flexibilities in rule interpretation can enable timely, care-driven responses; and peer networks among principals should be strengthened to exchange strategies for navigating bureaucratic constraints. Institutionalizing recognition practices—such as celebrating personal and professional milestones—can further enhance teachers’ sense of belonging and engagement. Policymakers should also consider granting school leaders greater discretionary authority to address urgent needs without delays caused by rigid processes.

Directions for future research: Priority avenues include longitudinal designs testing links between heartfelt leadership, burnout, retention, and student outcomes; comparative studies across governance models (centralized vs. decentralized) and cultural contexts; intervention studies that develop leaders’ social and emotional competencies and assess downstream effects; mixed-methods designs connecting leaders’ navigation strategies with climate and well-being indicators; and examinations of potential gendered dynamics in the enactment and reception of heartfelt leadership.

## Figures and Tables

**Table 1:** Themes, Sub-themes, and Sample Quotes.

Theme	Sub-Theme	Description	Sample Quote
Theme 1: Principals acknowledge the humanity of their teachers	Empathy and Emotional Awareness, Recognition of Significant Moments	Principals are seen not only as administrators but as individuals who recognize teachers’ humanity, attending to emotions and acknowledging meaningful milestones.	‘A principal should be present in both good and bad times, understanding what we go through and making us feel valued.’ (T1)
Theme 2: Principals prioritize relationships and go beyond formal duties	Providing Resources and Support, Building Informal Connections	Heartfelt leadership manifests in daily practices that exceed formal expectations, through proactive support and informal relational gestures.	‘Our principal paid competition fees from his own pocket.’ (T2)
Theme 3: Bureaucracy constrains but does not fully silence heartfelt leadership	Procedural Constraints Centralized Decision-Making and Lack of Autonomy	Even well-intentioned leaders are often thwarted by rigid bureaucracy, yet they still strive to enact care-driven practices where possible.	‘Even minor changes require multiple approvals.’ (A5)
Theme 4: Heartfelt leadership fosters motivation, resilience, and retention	Motivation and Engagement, Reduced Burnout and Emotional Resilience	Heartfelt leadership enhances teacher well-being, boosts motivation, and strengthens long-term commitment despite systemic barriers.	‘It reignited my passion for teaching.’ (T5)

## Data Availability

Due to ethical and privacy considerations, raw qualitative interview data are not publicly available. However, anonymized excerpts relevant to the findings may be provided by the corresponding author upon reasonable request, subject to ethics approval and data-sharing agreements.
